# Supplementary material for: Patterns of Intron Gain and Loss in Fungi
Source: PLoS Biol. 2004 Nov 30;2(12):e422. doi: 10.1371/journal.pbio.0020422 (PMC532390; doi:10.1371/journal.pbio.0020422)
Supplement: Table S1 — Also available at http://genes.mit.edu/NielsenEtAl/. (4.3 MB ZIP). [file pbio.0020422.st001.zip › NielsenEtAl/html/20.html]

AN3112.1.NCU01824.1.MG06695.1.FG05997.1


```
 CLUSTAL W (1.82) Multiple Sequence Alignments - Introns Inserted


Sequence 1: NCU01824.1	517 aa
Sequence 2: FG05997.1	516 aa
Sequence 3: MG06695.1	505 aa
Sequence 4: AN3112.1	532 aa
Alignment Length: 541 aa
Number Identitical Residues: 350 aa
Alignment Score (without introns) 16186


MG06695.1 	-------------------------MD1I2NIDVLIIGAGPTGLGAAKRLHHL0NGPSWL
NCU01824.1	------------------------MAD~I2NVDVLVIGAGPTGLGAAKRLNHI0NGPSWL
FG05997.1 	------------------------MAN~T2NVDVLVIGMGPTGLGAAKRLNHI0NGPSWL
AN3112.1  	MLSLARKTLNRVPSFQDILQGRMTHPD~I2SVDVLVIGAGPTGLGAAKRLNQI0NGPSWL
          	  : : .: .  .: ..  ..  :  :   .:***:** ***********::: ******

MG06695.1 	ILDSSEKAGGLASTDVTPEGF0LYDVGGHVIFSHYKYFDDCLDEALPKEDDWFTHQRISY
NCU01824.1	IVDANEKAGGLASTDVTPEGF~LYDVGGHVIFSHYKYFDDCLDEALPKADDWYTHQRISY
FG05997.1 	IVDSSDKAGGLASTDTTPEGF0LYDVGGHVIFSHYKYFDDCIDEALPKDDDWYTHQRISY
AN3112.1  	LVDSNETPGGLASTDVTPEGF0LYDVGGHVIFSHYKYFDDCINEALPKEDDWYEHQRISY
          	::*:.:..*******.***** *******************::***** ***: ******

MG06695.1 	VRYKNLWVPYPFQNNISMLPKEDQVKCIDGMIDAALEVRVANTKPKDFDEWIMRMTGEGV
NCU01824.1	VRYKGLWVPYPFQNNISMLPDEDKVKCVEGMIDAALEHRVANTKPKDFDEWILRNCGEGV
FG05997.1 	VRYKGLWVPYPFQNNISMLPKEDQVDCIEGIIDSALECRVANTKPKDFDEWIVRMMGEGI
AN3112.1  	VRCKGQWVPYPFQNNISMLPKEDQVKCIDGMIDAAIEHRVANTKPKDFDEWIVRMMGTGV
          	** *. **************.**:*.*::*:**:*:* **************:*  * *:

MG06695.1 	ADIFMRPYNYKVWAVPTTK0MQCQWLGERVAAPDVKLVTKNVILGKVAGNWGPNATFRFP
NCU01824.1	ANIFMRPYNYKVWAVPTTK0MQCEWLGERVAAPNVKLVTKNVILNKTAGNWGPNATFRFP
FG05997.1 	ANIFMRPYNYKVWAVPTKK0MQCQWLGERVAAPDAKLVTKNVILNKVAGNWGPNATFRFP
AN3112.1  	ADLFMRPYNYKVWAVPTTK0MQCAWLGERVAAPNVKAVTTNVILNKTAGNWGPNATFRFP
          	*::**************.* *** *********:.* **.****.*.*************

MG06695.1 	ARDGTGGIWIAVADTLPDEKKRFGKKGEVSKVDAENKVVTMADGTTIGYGKLISTMPVDH
NCU01824.1	ARDGTGGIWIAVSNTLPEKNKRYGKHGEVTKVDAEKKTVTLGDGTTVKYGKLINTMAVDH
FG05997.1 	ARDGTGGIWIAVAETIPKEKKRFGKQGEVTKIDGEKKIAYFADGSTISYNKLVNTMAVDH
AN3112.1  	ARDGTGGIWIAVANTLPKEKTRFGEKGKVTKVNPKNKTVTLGDGTTVGYQKLVSTMAVDY
          	************::*:*.::.*:*::*:*:*:: ::* . :.**:*: * **:.**.**:

MG06695.1 	LVEKMDRQDLVSLSQGLFYSSTHVIGIGLRGERPERIGDKCW~LYFPEDNCPFYRATVFS
NCU01824.1	LVEKMGNQELVSLSKGLYYSSTHVIGVGIRGERPERIGDKCW~LYFPEDNCPFYRATIFS
FG05997.1 	LAEKLGNQELMTLTKQLYYSTTHVIGVGIRGERPERIGDKCW~LYFPEDNCPFYRATIFS
AN3112.1  	LAEQIGDQELIGLTKQLFYSSTHVIGVGIRGSRPERIGDKCW0LYFPEDNCPFYRATIFS
          	*.*::. *:*: *:: *:**:*****:*:**.********** **************:**

MG06695.1 	NYSPYNQPDAEKKLPTMQLGDGSKPSSSEVKGGPYWSIMLEVSQSTMKPVDEENLLRDCI
NCU01824.1	NYSPYNQPEAGAKLPTLYKADGSKASSDEAKEGPYWSIMLEVSQSTVKPVDEENLLKDCI
FG05997.1 	NYSPYNQPQKDAKLPTLYLANGEKAASSEAKEGPYWSIMLEVSQSTMKPVDVENLLKDSI
AN3112.1  	NYSPYNQPDASKKLPTLQLADGSKPKNTEPQEGPYWSIMLEVSESSMKPVNIDTLLAESI
          	********:   ****:  .:*.*. . * : ***********:*::***: :.** :.*

MG06695.1 	QGLVNTDMIKADDEIVSTYHRKFDHGYPTPTLEREGVLKELLPKLQDMDIYSRGRFGSWR
NCU01824.1	QGLINTEMIKPEDEIVSTYHRKFDHGYPTPSLEREGVLKELLPKLQDMDIWSRGRFGSWR
FG05997.1 	QGLINTEMIKPEDEIVSTYHRAFDHGYPTPTLEREGVLKQVLPKLESMDILSRGRFGSWR
AN3112.1  	QGLVNTEMLKPGDEIVSTYHRRFDHGYPTPSLEREGALTQILPKLQSMDIWSRGRFGSWR
          	***:**:*:*. ********* ********:*****.*.::****:.*** *********

MG06695.1 	YEVGNQDHSFMLGVEAADHIVTGAVELTLNYPDFVNSRQNNERRLAQ---------QGFR
NCU01824.1	YEVGNQDHSFMLGVEAVDNIVTGAVELTLNYPDFVNGRQNTERRLAQSFVFGVKEDQSIP
FG05997.1 	YEVGNQDHSFMLGVEAVDAIHSGAVELTLNYPDFVNSRQNTERRLTQNFVFSTPQTNGIP
AN3112.1  	YEVGNQDHSFMLGVEAVDNIVNGAVELTLNYPDFVNGRQNTERRLVDG-------AQAFA
          	****************.* * .**************.***.****.:.        :.: 

MG06695.1 	PEANA1--
NCU01824.1	ARSAL~PN
FG05997.1 	SHSKA~Q-
AN3112.1  	KNKAQ~--
          	 .
```
